# Social or Economic Goals? The Professional Goal Orientation of Students Enrolled in STEM and Non-STEM Majors in University

**DOI:** 10.3389/fpsyg.2019.02065

**Published:** 2019-09-13

**Authors:** Ilka Wolter, Lisa Ehrtmann, Tina Seidel, Barbara Drechsel

**Affiliations:** ^1^Competencies, Personality, Learning Environments, Leibniz Institute for Educational Trajectories, Bamberg, Germany; ^2^Friedl Schöller Endowed Chair for Educational Psychology, School of Education, Technical University of Munich (TUM), Munich, Germany; ^3^Institute of Psychology, University of Bamberg, Bamberg, Germany

**Keywords:** STEM, goal orientation, latent profile analysis, university students, gender stereotypes, meaning of work

## Abstract

Various studies try to disentangle the gender-specific competencies or decisions that lead to a career in a STEM field and try to find a way to encourage more women to pursue this kind of career. The present study examines differences in the meaning of work (i.e., their professional goal orientation) of students who are enrolled in STEM or non-STEM programs in tertiary education. Based on the background that gender stereotypes associate women and men with communal or agentic roles respectively, we expected that women in STEM subjects differ in their professional goal orientation from women in non-STEM programs. More precisely, women who are enrolled in a STEM major are expected to be less oriented to social and communal goal orientations than women in non-STEM university programs. In a sample of 5,857 second-year university students of the German National Educational Panel Study, three profiles of professional goal orientation were confirmed in a latent profile analysis. As expected, women were more oriented toward social aspects of occupations, whereas men more likely belonged to a profile with high importance for economic aspects of occupations. Moreover, students enrolled in STEM programs more likely belonged to the profile of economic goal orientation. There was, however, no interaction of gender and STEM program: Women in STEM fields did not differ in their occupational goal orientation from women enrolled in non-STEM programs. Based on these findings and on a goal congruity perspective, future interventions aiming at overcoming the underrepresentation of women in STEM fields should consider the individual meaning of work and the goals that are associated with STEM occupations.

## Theoretical Background

Gender segregation in the labor market and in university majors is a widely known and consistent pattern of previous empirical research (e.g., Leuze and Strauß, 2009; Bechmann et al., 2012; Ochsenfeld, 2012; Hausmann and Kleinert, 2014), showing in detail that women are especially underrepresented in STEM fields (i.e., science, technology, engineering, and mathematics). This segregation is particularly concerning because STEM fields are mostly characterized by higher prestige and income (e.g., Oh and Lewis, 2011). However, research also showed that the mechanisms of this gap need further investigation and differentiation, in particular with respect to STEM related subdisciplines. The gender ratio is by far not as consistent as it seems at a first glance (c.f. Gisler et al., 2018). An analysis of the official statistics of first-year university students in the winter term of 2017/2018 for Germany, as a highly STEM-industrialized western country, showed the unequal proportion of women and men who are enrolled in different subdisciplines within STEM majors: While women are in fact overrepresented in subjects such as biology (62.0%), pharmacy (68.6%), or architecture (57.9%), they are decidedly underrepresented in subjects like physics (28.7%), engineering (22.3%), or computer sciences (21.1%) (German Federal Bureau of Statistics, 2018).

Not only recently, there is an international call towards researchers and policy-makers to focus on developing interventions to encourage women to engage in STEM majors in universities and to follow careers in these domains (c.f. Liben and Coyle, 2014). It is important to mention that the debate also emphasizes gender differences in abilities as an explanation for gender gaps in STEM fields. Gender differences in domain-specific competencies such as mathematics or reading are consistently shown in empirical research (see PISA 2015; OECD, 2016). Furthermore, boys are on average better at spatial tasks than girls, who are in turn better at verbal tasks (for a review, see Spelke, 2005). However, most studies only find small differences and the current discussion focuses more on the proposition of the gender similarities hypothesis (Hyde, 2005, 2014).

The most recent PISA study in 2015 placed a particular focus on the science competence of 15-year-olds in Germany and investigated whether students in adolescence already show aspirations towards STEM occupations (for Germany: Schiepe-Tiska et al., 2016b). The findings revealed for the example of Germany that 27% of boys, yet, only 18% of girls indicated that they would consider pursuing a career in science at the age of 30. The study further confirmed that there are differences with respect to certain subdisciplines. Boys are more interested in mathematics and information technology, whereas girls are more interested in health related occupations within STEM fields (Schiepe-Tiska et al., 2016a). The results also confirmed that science competence is rather unimportant in predicting adolescents’ aspiration to pursue a STEM career, but instead their instrumental motivation and their enjoyment of science tasks were positively related to their aspiration toward STEM (Schiepe-Tiska et al., 2016a,b).

Thus, in the recent scientific discussion, gender differences in domain-specific competencies are not considered sufficient in fully explaining differences in choices for STEM careers, and researchers focus more often on affective-motivational and other non-cognitive explaining factors.

### Psychological Mechanisms for Educational and Occupational Choices Into Science Technology Engineering Mathematics Fields

In looking more closely into factors that might be able to explain differences in choosing a STEM vs. non-STEM occupation or study course, psychological research provides a variety of explanations with respect to educational decisions and motivation as well as stereotypes and images of STEM subjects.

Gender-specific educational decisions and motivation are often displayed in the wide known metaphor of the “pipeline issue” (Clark Blickenstaff, 2005; Maltese and Tai, 2011; Cannady et al., 2014). This line of discussion describes the phenomenon that fewer girls than boys choose to study STEM subjects already in secondary school which then again leads to less women who decide to study STEM subjects in university or to work in STEM-related occupations (c.f. for Germany: German Federal Bureau of Statistics, 2018). Moreover, there is also the issue of the “leaky pipeline” (Alper, 1993) describing that, in addition to the lower proportion of women who start a STEM career, they were also more likely to drop-out during the course of an education within a STEM field. The pipeline-metaphor is criticized not only for suggesting a linear path within a STEM career and for neglecting the role of gatekeepers in this process but also for providing a seemingly easy fix for policy-makers (Cannady et al., 2014). Also, a report by the Committee on Barriers and Opportunities in Completing 2-year and 4-year STEM degrees appointed by the National Academies of Sciences, Engineering, and Medicine (2016) points out that there is a variety of paths within STEM careers and that it is not advisable to propose only a linear route. The report also states that this inaccurate image of STEM careers is a reason why most efforts to intervene fail because it does not acknowledge the more complex ways and challenges that students face within their STEM education. In that same respect, previous research also argues that explanations for the high attrition rate in STEM majors in general are a lack or loss of interest in STEM subjects, poor teaching of STEM faculty, or inadequate advising and help with academic difficulties (e.g., Seymour, 1992). Therefore, without the intention to simplify the obstacles for women to choose a STEM career, from our point of view, the metaphor still quite well symbolizes the smaller probability to enter a STEM field as well as the larger drop-out for women in STEM careers; however, it does not provide an extensive image, especially with the aim to derive intervention programs.

In fact, the reasons for these drop-outs are manifold: Studies, for example, confirmed external factors such as a discrimination against women in hiring processes within STEM fields (e.g., Moss-Racusin et al., 2012). It is, however, also often discussed that there are internal factors which lead women to opt out of a STEM career, for example, the difficult compatibility of different roles with respect to a reasonable work-family-balance within those fields (e.g., Blair-Loy, 2003; Diekman et al., 2010). Seymour (1992) reported that more than half of the students who switched from STEM majors to non-STEM majors indicated the rejection of STEM careers or the associated lifestyle, respectively, as a concern that contributed to their decision. As a consequence, there is a small number of women who work in or study STEM fields in tertiary education in Germany (German Federal Bureau of Statistics, 2018). One prominent theory that is often consulted is the expectancy-value theory (c.f. Wigfield and Eccles, 1992) which describes motivational and self-evaluative aspects of career decisions that are influenced by individual characteristics, but also formed by socialization, and expectations from teachers (e.g., Beilock et al., 2010; Upadyaya and Eccles, 2014) or parents (e.g., Räty et al., 2002; Tenenbaum and Leaper, 2003; Lindberg et al., 2008). Additionally, according to this theory, the value that is assigned to certain subjects or fields of occupation plays an important role for educational decisions. For example, Lent et al. (1994) argued in the social-cognitive career theory that individuals’ self-efficacy or expected outcomes are relevant for the development of occupational interests which in turn are related to occupational success, aspirations, and decisions (Lent et al., 1994, 2010). Lent et al. (2018) conducted a meta-analysis combining the findings of 143 studies and showed that perceived support and perceived barriers are in general relevant for individuals’ choice goals, but also that in particular for women perceived barriers are relevant for their outcome expectations (Lent et al., 2018).

Another theoretical angle to describe the phenomenon of underrepresentation of women in STEM fields is related to the stereotypes and images that are associated with these domains. A lot of empirical studies have confirmed the gender-science and gender-math stereotypes in implicit association tests according to which mathematics and sciences are perceived as male-stereotyped domains (e.g., Nosek et al., 2009; Plante et al., 2009; Steffens et al., 2010; Cvencek et al., 2011; Steffens and Jelenec, 2011; Passolunghi et al., 2014). Moreover, empirical evidence shows that those stereotypes also predict gender differences in science and mathematical achievements (e.g., Nosek et al., 2009). Additionally, alternative studies focused on the perceived images of STEM subjects and the self-to-prototype matching strategy (c.f. Niedenthal et al., 1985). Previous research shows, for example, that students who indicated physics as their favorite subject are perceived as intelligent, but at the same time as unpopular and unattractive (Hannover and Kessels, 2004).

### Goal Congruity Approach

According to the goal congruity theory, individuals strive to live in congruence to their goals and to the perceived expectations of their environment; therefore, an individual’s communal goal orientation might discourage them from pursuing a STEM career (c.f. Diekman and Steinberg, 2013; Diekman et al., 2015). Early on, Bakan (1966) proposed that there are agentic and communal motivations, and these motivations were stated as relevant to social judgment and self-concepts (c.f. warmth-competence distinction by Fiske et al., 2007). Even though communal traits are valued in men and women, gender norms particularly associate them to women (e.g., Diekman and Goodfriend, 2006). Against the background of previous research on gender stereotypes (i.e., the association of attributes, traits, tasks to either gender group, c.f. Eagly, 1987; Eagly and Wood, 2016), women’s and men’s meaning of work or their professional goal orientation should differ according to these stereotypes. Gender stereotypes are defined as how women and men are perceived and what is expected of them (i.e., descriptive and prescriptive component of stereotypes, Eagly, 1987). Research showed that gender stereotypes are categorized along the dimensions of “people-things” (Su et al., 2009) or “communal-agentic traits” (e.g., Abele, 2003; Abele and Wojciszke, 2007; Fiske et al., 2007). These stereotypes describe that women are more likely associated to activities (including occupations) related to interactions with people, caring, and taking social responsibilities (e.g., as a communal role). Men, however, are stereotypically associated to handling “things,” securing the financial situation of the family (e.g., breadwinner), and being in charge (e.g., as an agentic role) (e.g., Eagly and Wood, 2016). Therefore, individuals’ occupational goals and their meaning of work is expected to differ for women and men: Women are expected to be oriented toward social aspects and a satisfactory work-family balance, yet, the professional goal orientations of men should reflect more competitive, individualistic, and economic goals.

The current study focuses on a particular aspect of occupational goal orientation, namely individuals’ meaning of work. Against the empirical and theoretical background, students’ meaning of work (MOW) could be of particular interest in explaining the gender-gap in STEM fields. Individuals’ meaning of work are described by the significance and value that is associated to work and occupations (c.f. Ruiz-Quintanilla, 1991; Claes and Ruiz-Quintanilla, 1993). We expect that students in STEM and non-STEM programs in university might be differentiated with respect to their goal orientation according to gender stereotypes. Students in STEM programs are aspiring to higher income and secure and prestigious jobs (Oh and Lewis, 2011), whereas the more heterogeneous group of students in non-STEM majors might be characterized by placing more importance to social goals or expressive aspects of occupations. Furthermore, women should in general strive for a comfortable work situation and flexible hours to balance their work with their family life. Men, however, should be aspiring more economic security according to the breadwinner model (c.f. social role theory, Eagly and Wood, 2016). We further expect that there is an interaction with students’ gender: Women who decide to study a STEM subject should be less in line with female stereotypical goals such as care-taking and societal or social responsibilities than women who decide to choose a non-STEM major. Men, however, should probably show different goal orientations when they pursue a communal career (c.f. Croft et al., 2015) than men who pursue STEM subjects. Yet, since non-STEM occupations are very heterogeneous and not only consist of communal occupations, we do not expect large differences for men. Since STEM fields are also related to higher income and prestige (Oh and Lewis, 2011) students’ socio-economic background should explain interindividual differences in students’ professional goal orientations. To sum up, the current study aims at a comparison of students in STEM and non-STEM programs by examining differences in their professional goal orientation.

## Research Aims and Hypotheses

Previous studies focused on the impact of competencies, motivation, or expectations on individuals’ decisions to pursue a career in a STEM field. To add to the various aspects of explaining the underrepresentation of women in STEM, we argue that individuals’ professional goal orientation (i.e., meaning of work) might also be related to educational and occupational decisions and tenacity. This should be true especially for women whose major subjects are within STEM fields and therefore more often associated to men. Therefore, our hypotheses were as follows:

(1a) Specific profiles of students’ professional goal orientation (i.e., meaning of work) can be detected. Next to a profile with overall high and a profile with overall low goal orientations, we hypothesize two specific profiles that are described as either focused on social, and well-being aspects of working (c.f. communal role) or on economic and autonomy aspects of working (c.f. agentic role). We further hypothesize that (1b) women more likely belong to the profile of high social goal orientations, whereas men more likely belong to the profile of high economic goal orientations.

(2) Students who pursue a career in a STEM field as compared to students in non-STEM majors differ in their professional goal orientations. (2a) Students who are enrolled in non-STEM majors are more likely members of a profile with social and well-being aspects of working, whereas (2b) students who are enrolled in STEM fields more likely belong to the profile of economic or autonomy aspects of working. Furthermore, we expect (2c) an interaction of students’ gender and their study major. Women in STEM majors should less likely belong to a profile of social goal orientations than women in non-STEM majors.

## Materials and Methods

### Sample

In the current study, we did a secondary data analysis with data from a sample of *N =* 13,113 university students in their first to second academic year (i.e., wave 1; winter term 2010/2011; starting cohort (5) of the German National Educational Panel Study (NEPS; Blossfeld et al., 2011). The German National Educational Panel study provides longitudinal data from six representative starting cohorts within a multi-cohort sequence design (starting with a birth cohort and up to adulthood). In this study, we used data from the NEPS starting cohort five of university students in their first academic year. For registered researchers, the data are available as Scientific Use File and more information on the design, cohort information, and the measurements are documented on www.neps-data.de. We aimed at identifying first-year university students who were enrolled in their majors in either STEM or non-STEM fields. In order to distinctly identify the STEM-related subjects of the students, we excluded 4,252 students (32.4%) of the sample who studied toward a teaching degree (either bachelor degree or state examination for teaching degree) because most of them have a combination of two to three subjects which are not necessarily within the same categories with respect to STEM and non-STEM. However, this exclusion criterion was not based on any reasons implying less relevance of this particular group of students. We surely acknowledge the major role (prospective) teachers play in modeling (gender-typical) behavior, especially in science (c.f. Stout et al., 2011). We further excluded students who are enrolled in a university of applied sciences (*n =* 2,967; *n =* 5 students had missing values on the type of university, *n =* 3 students studied abroad or indicated to have no university). We argue that there is no coherent theoretical outline to include both types of institutions (i.e., university or university of applied sciences) for this research question since universities of applied sciences are more directly oriented toward the labor market and also show differences with respect to the provided study majors; for example, universities of applied sciences provide more courses in engineering than universities.

The sample consists of *n =* 5,883 students who are enrolled in a university in Germany (*n =* 3 students had missing values on their major program at university). We used the information about students’ first major subject in university, yet, *n =* 183 students indicated to study in a bachelor program with two major subjects. From this subsample, we excluded *n =* 29 students because their first and second major subjects were not coherently both in either STEM or non-STEM fields. Students’ minor subjects were not taken into account. The final sample for our analyses was *n =* 5,857. The students were asked about their goal orientation (i.e., meaning of work) in their second year in university (i.e., wave 3, summer term 2012).

### Research Instruments

#### STEM and Non-STEM Program

First-year students’ main study majors in university were categorized as STEM or non-STEM majors according to the categorization of major subjects in the winter term of 2010 of the German Federal Bureau of Statistics (2018) (*n =* 3 missing values on first major subject). This categorization subsumes STEM fields for all subjects in the area of mathematics and science (mathematics and science in general, mathematics, physics, astronomy, chemistry, pharmacy, biology, geological science, and geography) as well as engineering (engineering in general, mining industry/metallurgy, mechanical engineering/process engineering, electrical engineering/ information engineering, traffic engineering/nautical science, architecture/interior design, city and regional planning, construction engineering, surveying and mapping, industrial engineering, computer sciences, and materials sciences/materials engineering). We categorized the students’ subjects accordingly. Students were more often enrolled in non-STEM majors (*n =* 3,597; 61.4%) than in STEM majors (*n =* 2,257; 38.6%) which is comparable to the proportion of students in STEM majors (38.0%) and non-STEM majors in Germany in the winter term 2010/2011 (reference: students in first semester at university; German Federal Bureau of Statistics, 2011, p. 34). Of the students enrolled in STEM majors 42.3% (*n =* 954) were enrolled in engineering, whereas 57.7% (*n =* 1,303) were enrolled in mathematics and science.

In reference to the group of students in non-STEM majors, the largest groups were students enrolled in law, economic, and social sciences (46.9%, *n =* 1,688) followed by students in language and cultural studies (29.5%, *n =* 1,061). The smaller groups were students enrolled in medicine and health-related sciences (15.3%, *n =* 552), agricultural, forestry, and nutritional sciences (2.5%, *n =* 90), arts (3.4%, *n =* 123), sports (1.3%, *n =* 47), and veterinary medicine (1.0%, *n =* 36). This distribution of first-year students is comparable to the expected proportion of students in the subgroup of non-STEM majors who were enrolled in winter term 2010/2011 at German universities (c.f. German Federal Bureau of Statistics, 2011; students only in non-STEM majors were enrolled as follows: 54.0% law, economic and social sciences; 28.0% language and cultural studies; 7.2% medicine and health related sciences; 3.3% agricultural, forestry and nutritional sciences; 5.6% arts; 1.5% sports; and 0.4% veterinary medicine).

##### Students’ Professional Goal Orientations

Students’ professional goal orientations (i.e., meaning of work) (c.f. Ruiz-Quintanilla, 1991) describe the importance of goals and activities associated to occupations independent of the individual’s current situation. These occupational goal orientations were measured on a six-point scale from “1 not important at all” to “6 very important”. The theoretically expected six subscales of this questionnaire were (1) learning (e.g., “Opportunity to learn new things”, two items), (2) social orientation (e.g., “A work that is useful for the society”, three items), (3) autonomy (e.g., “Own decision making competence”, two items), (4) economic aspects (e.g., “Good chances to move up the career ladder”, three items), (5) comfort aspects (e.g., “Pleasant working hours”, two items), (6) expressive aspects (e.g., “Diverse tasks”, four items). Before including these dimensions into the latent profile analysis, however, we checked the factor structure in a confirmatory factor analysis. The model fit for the original factor model was not satisfactory with *χ*^2^ *=* 4252.40, df = 89, *p* < 0.001, CFI = 0.80; TLI = 0.73, RMSEA = 0.089 and hinted to a problem in the dimension of expressive aspects of goal orientations. Therefore, we conducted an exploratory factor analysis to check the empirical validity of these dimensions.

#### Student’s Economic Situation

Students’ economic situation was measured *via* a question regarding the perceived difficulties to provide things or to pay fees for the study course (“How hard is it for you and your family to pay for the things that you need for your academic studies, for example, travel expenses, books, or tuition fees?”). This question was measured on a five-point scale from “1, very difficult” to “5, very easy” and afterwards recoded so that higher values indicated a higher financial hardship of the according student. Students indicated on average a medium burden due to financial issues, *M =* 2.51, SD *=* 0.97, range 1–5, missing values *n =* 14.

### Analysis Plan

In order to test the hypotheses, an exploratory factor analysis as well as a latent profile analysis were conducted using Mplus Version 8 (Muthén and Muthén, 2017). The preparation of the data set and some of the preliminary descriptive analyses were conducted in SPSS. First, the exploratory factor analysis with the scale goal orientation was conducted to empirically test the dimensions of this construct. Second, the latent profile analysis was conducted with a comparison of latent profile solutions from two to five profiles. For the latent profile analysis, the latent profile indicators were the factors of students’ professional goal orientations. To test the profile specific hypotheses, the automatic three-step method implemented by Mplus through the R3STEP command was used (c.f. Asparouhov and Muthén, 2014). Afterwards, the thereby established profile memberships were fixed and used in a multinomial logistic regression as dependent variables with auxiliary variables (i.e., the predictor variables). Independent variables in this analysis were students’ gender (male −0.5; female 0.5) and whether students were enrolled in a STEM major or a non-STEM major (non-STEM −0.5; STEM 0.5) as well as the interaction of students’ gender and the STEM vs. non-STEM programs. Furthermore, the control variable students’ economic situation (grand-mean centered) as well as the interaction of economic situation and STEM major was included in the analysis. The Mplus syntax and model outputs for the exploratory factor analysis and the latent profile analyses are available under: https://osf.io/k86ny/.

Cases with missing values on either dependent or independent variables were excluded from the multinomial logistic regression analysis but included in the latent profile analysis. However, there were only *n =* 17 missing cases (<1%) on the predictor variables; therefore, listwise deletion should not lead to biased results in this analysis.

## Results

### Preliminary Analyses

#### Sample Descriptives

In the first step, we compared the two groups of students with respect to the gender distribution and their economic situation. As expected, 74.3% of women were enrolled in a non-STEM major as compared to STEM major subjects, whereas 54.6% of men were enrolled in a STEM major as compared to non-STEM programs. The unequal proportion of women and men in STEM majors was statistically significant, *χ*^2^ *=* 512.71, df *=* 2, *p < 0*.001.

Furthermore, student’s economic situation differed between the two groups, however, with only a small effect size: Students in STEM majors reported slightly less burden (*M =* 2.46, SD *=* 0.95) than students who were enrolled in non-STEM majors (*M =* 2.55, SD *=* 0.98), *t*_(5838)_ = 3.42, *p =* 0.001, *d =* 0.09.

#### Exploratory Factor Analyses

In the second step, comparative exploratory factor analyses were conducted to test the subdimensions of students’ occupational goal orientation. The model fits for the factor solutions of a unidimensional up to a six-factor model were compared by the conventions described for example in Hu and Bentler (1999). The comparison of model fit information (as displayed in Table 1) suggested a five- or six-factor solution for this scale. Yet, the subdimensions between the five- and six-factor models only differed slightly and did not add meaningful information to the goal orientations because none of the items loaded substantially better on the sixth factor. Therefore, the five-factor model with RMSEA = 0.044; CFI = 0.973, and TLI = 0.936 was chosen for the following analysis.

**Table 1:** Model comparison of one- to six-factor solution (exploratory factor analyses) for students’ goal orientations.

	AIC	BIC	RMSEA	CFI	TLI
1-factor	244490.23	244810.65	0.123	0.559	0.491
2-factors	240593.58	241014.13	0.101	0.746	0.658
3-factors	237731.97	238245.98	0.074	0.884	0.814
4-factors	236556.24	237157.02	0.058	0.941	0.886
5-factors	235891.88	236572.77	0.044	0.973	0.936
6-factors	235671.32	236425.64	0.038	0.984	0.952

The five dimensions are labeled as oriented toward (1) social, (2) psychosocial health, (3) economic, (4) autonomy, and (5) motivational goal orientations. The *social factor* (measured by three items; Cronbach’s alpha = 0.71) is described by the importance of useful work, of helping others, and doing something meaningful in work. The *psychosocial health factor* (measured by five items; Cronbach’s alpha = 0.59) comprises a high significance of a good workplace climate, good working hours, and physical working conditions as well as secure employment, and a high match of skills and demands in the workplace. The *economic factor* (measured by two items; Cronbach’s alpha = 0.69) combines the relevance of a good payment and opportunities for advancements. The *autonomy factor* (measured by three items; Cronbach’s alpha = 0.72) is described by the importance of being independent, of having the authority to decide, and of being in charge. The *motivational factor* (measured by three items; Cronbach’s alpha = 0.65) includes the importance of learning new things, facing manifold tasks, and having interesting work.

#### Descriptive Analyses of Students’ Goal Orientations

The means and standard deviations of the factors of the construct goal orientation (i.e., meaning of work) with the five-factor solution are displayed in Table 2 for the overall sample and separate for students in STEM and in non-STEM majors.

**Table 2:** Descriptive analyses of subdimensions of students’ goal orientations.

Occupational goal orientation	sample (*N =* 5,857)		Non-STEM (*n =* 3,597)		STEM (*n =* 2,257)		Mean difference (df *=* 5,852)		
	*M*	SD	*M*	SD	*M*	SD	*t*	*p*	*d*
Social (three items)	4.78	0.80	4.88	0.80	4.63	0.79	11.80	<0.001	−0.32
Psychosocial health (five items)	4.69	0.63	4.70	0.63	4.66	0.64	2.59	0.010	−0.07
Economic (two items)	4.68	0.87	4.65	0.88	4.72	0.85	3.53	<0.001	0.09
Autonomy (three items)	4.29	0.80	4.36	0.80	4.19	0.80	7.67	<0.001	−0.21
Motivational (three items)	5.20	0.59	5.24	0.59	5.16	0.59	4.97	<0.001	−0.13

In general, students in non-STEM majors show higher goal orientations across almost all dimensions, except economic goals which were higher for students enrolled in STEM majors. The largest difference between students in non-STEM and STEM majors existed in the dimension of social goal orientation [*t*_(5852)_ *=* 11.80, *p <* 0.001, *d =* −0.32], followed by autonomy goal orientation [*t*_(5852)_ *=* 7.67, *p <* 0.001, *d =* −0.21], motivational goal orientation [*t*_(5852)_ *=* 4.97, *p <* 0.001, *d =* −0.13], and rather small differences in students’ economic goal orientation [*t*_(5852)_ *=* 3.53, *p <* 0.001, *d =* 0.09], and the psychosocial health factor of the goal orientations [*t*_(5852)_ *=* 2.59, *p =* 0.010, *d =* −0.07]. The intercorrelations of the subdimensions of the construct are displayed in Table 3.

**Table 3:** Intercorrelation of subdimensions of students’ goal orientations.

	1	2	3	4	5
Social (1)	1.00	0.32	0.01	0.25	0.40
Psychosocial health (2)		1.00	0.38	0.28	0.28
Economic (3)			1.00	0.36	0.13
Autonomy (4)				1.00	0.37
Motivational (5)					1.00

All subdimensions of the five-factor model of students’ goal orientation are moderately correlated with each other (between *r =* 0.13 for economic and motivational factor to *r =* 0.40 for social and motivational factor); additionally, there is a zero correlation between the social and the economic factor of students’ goal orientation (*r =* 0.01).

### Results of Latent Profile Analysis

First, simple latent profile analyses with profiles differing from three to six were conducted to determine if the presumed four-profile solution was acceptable. According to the comparison of Akaike information criterion (AIC) and Bayesian information criterion (BIC) (e.g., Nylund et al., 2007) and considering the Vuong-Lo-Mendell-Rubin likelihood ratio test (Vuong, 1989; Lo et al., 2001), the three-profile model had the overall best acceptable fit (AIC = 61308.17, BIC = 61455.03, VLMR = 770.32, *p =* 0.015) compared to a two-profile model (AIC = 62066.49, BIC = 62173.30, VLMR = 2763.74, *p <* 0.001), to a four-profile model (AIC = 60753.40, BIC = 60940.31, VLMR = 566.78, *p =* 0.209), and to a five-profile model (AIC = 60412.26, BIC = 60639.22, VLMR = 353.14, *p = 0*.350). The Bayesian information criterion (BIC), the Akaike information criterion (AIC), the Vuong-Lo-Mendell-Rubin likelihood ratio test value, and the entropy of each latent profile is displayed in Table 4.

**Table 4:** Model fits of two- to five-profiles solutions from latent profile analyses of students’ goal orientations.

	AIC	BIC	VLMR	Entropy
2-profiles	62066.49	62173.30	*p <* 0.001	0.588
3-profiles	61308.17	61455.03	*p =* 0.015	0.613
4-profiles	60753.40	60940.31	*p =* 0.209	0.654
5-profiles	60412.26	60639.22	*p =* 0.350	0.657

*VLMR, Vuong-Lo-Mendell-Rubin likelihood ratio test*.

**Figure 1:**
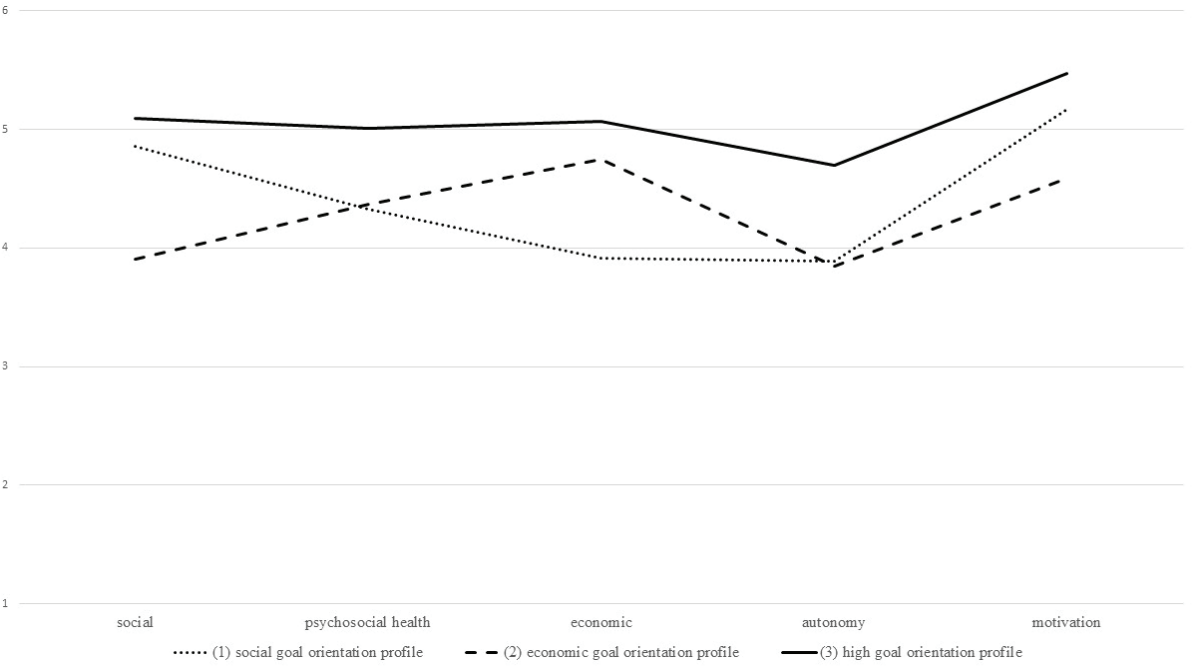
Latent profile analysis of students’ goal orientation: three-profile solution.

The substantial decrease in AIC and BIC as well as a slightly better entropy (albeit it still points to less distinguishable profiles) results in accepting the three-profile solution. Even though the four-profile solution shows slightly better fit indices with respect to AIC and BIC, the sizes of the profiles become very small (one profile consists of *n =* 184 individuals; 4.8% of the sample). The results of the three-profile latent model are displayed in Figure 1.

The findings of the latent profile analysis resulted in one general profile of students’ goal orientations and two more or less specific goal orientation profiles, partially confirming our hypothesis 1a. The general profile of goal orientation is characterized by a profile with high overall goal orientation (“high goal orientation profile”; *n =* 3,144 students, 53.7% of the sample). The findings did not confirm a profile with relatively low goal orientation since students showed in general rather high goal orientations in all aspects of occupational goals. Furthermore, students in all profiles barely differed in their motivational goal orientation. The specific goal orientation profiles included one “social goal orientation profile” (*n =* 1,611 students, 27.5% of the sample) with relatively high importance toward a meaningful job for society and helping others as well as by trend toward the security of employment and pleasant working conditions in the workplace. The second specific goal orientation profile was characterized by high importance to economic goals as compared to the other goals and was therefore labeled as the “economic goal orientation profile” (*n =* 1,102 students, 18.8% of the sample).

The percentages of female and male students as well as students in non-STEM and STEM program within the three profiles of goal orientation are displayed in Table 5.

**Table 5:** Frequencies (in percent) for the allocated profiles of students’ goal orientation separate for female and male students as well as for students in non-STEM and STEM programs.

	Female students	Male students	Non-STEM program	STEM program
Social goal orientation profile	57.1	42.9	63.7	36.3
Economic goal orientation profile	40.7	59.3	51.6	48.4
High goal orientation profile	60.0	40.0	63.7	36.3

### Results of Multinomial Logistic Regression Analysis

The results of the multinomial logistic regression model are displayed in Table 6. As expected in hypothesis 1b, women were more likely than men in the social goal orientation profile (*b =* 0.85, SE *=* 0.12, *p <* 0.001, OR = 2.33) and in the high goal orientation profile (*b =* 0.91, SE *=* 0.08, *p <* 0.001, OR = 2.48) in reference to the economic goal orientation profile. As a consequence, men were more likely in the economic goal orientation profile relative to the social goal orientation profile and relative to the high goal orientation profile. Students who are enrolled in a STEM program were more likely in the economic goal orientation profile than in the social goal orientation profile (*b =* 0.54, SE *=* 0.19, *p* = 0.004, OR = 1.72) and less likely in the high goal orientations (*b =* −0.42, SE *=* 0.13, *p =* 0.001; OR = 0.66), confirming our hypothesis 2b. That means that students who are enrolled in non-STEM programs were more likely in the social goal orientation profile and in the high goal orientation profile, confirming our hypothesis 2a. Students in STEM or non-STEM programs had equal probabilities to belong to the social goal orientation profile in reference to the high goal orientation profile (*b =* 0.12, SE *=* 0.14, *p =* 0.396, OR = 1.13). Overall, contrary to our hypothesis, there were no significant interactions of students’ gender and them being enrolled in a STEM field, hypothesis 2c was therefore not confirmed. Concerning the control variable, there were no significant effects of student’s economic situation with respect to the probability for either profile of goal orientation (e.g., social vs. economic profile of goal orientation: *b =* 0.03, SE *=* 0.07, *p =* 0.636, OR = 1.03). Furthermore, the interaction of students’ financial situation and STEM major was also not relevant for their profile of goal orientation (e.g., social vs. economic profile of goal orientation: *b =* 0.10, SE *=* 0.14, *p =* 0.481, OR = 1.11).

**Table 6:** Results of multinomial logistic regression analysis.

Ref. Profile			Estimate	SE	*p*	OR
**Social**						
	Economic	Intercept	−0.29			
		Economic situation	0.03	0.07	0.636	1.03
		STEM	0.54	0.19	0.004	1.72
		Gender	−0.85	0.12	<0.001	0.43
		Economic × STEM	0.10	0.14	0.481	1.11
		Gender × STEM	−0.02	0.25	0.922	0.98
	High	Intercept	0.59			
		Economic situation	0.07	0.05	0.160	1.07
		STEM	0.12	0.14	0.396	1.13
		Gender	0.06	0.09	0.501	1.06
		Economic × STEM	0.02	0.11	0.840	1.02
		Gender × STEM	−0.21	0.17	0.223	0.81
**Economic**						
	High	Intercept	0.88			
		Economic situation	0.04	0.04	0.377	1.04
		STEM	−0.42	0.13	0.001	0.66
		Gender	0.91	0.08	<0.001	2.48
		Economic × STEM	−0.08	0.11	0.461	0.92
		Gender × STEM	−0.23	0.18	0.208	0.79

*Gender × STEM, interaction term between STEM and gender; Economic × STEM, interaction term between STEM and economic situation; STEM, lower value non-STEM; Gender, lower value male; OR, odds ratio*.

## Discussion

Against the background of women’s underrepresentation in STEM fields (e.g., German federal employment office, 2018), the aim of this study was to investigate latent profiles of students’ occupational goal orientations (i.e., their meaning of work) in their second year in university. Furthermore, we aimed at comparing the goal orientations of two groups of students who were enrolled either in a STEM or in a non-STEM study program in higher education. The results first confirmed that there were differentiated profiles of students’ professional goal orientations. In more detail, there were three profiles of students’ goal orientations along the five dimensions of goal orientations labeled (1) social, (2) psychosocial health, (3) economic, (4) autonomy, and (5) motivational factors. First, there was one profile that was characterized by very high occupational goal orientation in general. Additionally, two profiles that are more specific were confirmed. One profile was characterized by a relatively high orientation toward social goals and well-being aspects combined with low orientation toward economic goals. The other specific profile was characterized by a relatively high orientation toward economic goals combined with rather low orientations toward social goals. In general, students (and therefore profiles) did not differ much in their motivational goal orientations. Consequently, the entropy of our profile solution was not very high, because at least this dimension, but also by trend the dimensions psychosocial health, and autonomy did not sufficiently differentiate between students’ profiles. Overall, it is likely that there was less variation in the motivational factor because students are in general highly motivated to learn new things and to challenge themselves with diverse tasks when entering higher (tertiary) education.

As expected, gender does make a difference: Women were on average more than two times as likely in the social or high goal orientation profile than in the economic goal orientation profile, whereas men were more likely members of the economic goal orientation profile. This is in line with previous research showing that women are more associated with communal roles and taking care of others (e.g., Abele, 2003; Eagly and Wood, 2016). These aspects are reflected in the social dimension of the goal orientations, which include doing a meaningful work, and helping others. Men, however, are more associated with agentic roles (Abele and Wojciszke, 2007; Fiske et al., 2007) which are linked to being in charge, being the family breadwinner (c.f. social role theory, Eagly, 1987), and being competitive. This agentic role—but especially the breadwinner role—is much more reflected in the economic goal orientation profile, even though the autonomy dimension only shows a slightly higher magnitude. It is plausible to assume that entering university is in general associated with high independence and autonomy and that this is why we did not find meaningful differences in this dimension.

However, contrary to our hypotheses, we did not find an interaction of student’s gender and STEM affiliation. Women in STEM did not differ in their membership to either of the profiles from women in non-STEM fields. The same applies to men: Men in STEM fields showed equal probabilities for the latent profiles as men in non-STEM fields. This is particularly important with respect to recent efforts and campaigns to get women involved in STEM fields (c.f. Liben and Coyle, 2014; Diekman et al., 2015). It seems from our study that women show in general a relatively higher orientation toward communal goals (e.g., Abele, 2003) which is in line with research on gender stereotypes (e.g., Fiske et al., 2007). Women in STEM programs, however, do not show a different pattern with respect to their goal orientations than their peers in non-STEM study programs according to our study. One explanation might be, however, that the results are not definitive and clear to interpret with respect to our hypothesis because many subjects are included in the broader area of STEM fields that are considered female gender-typed, such as biology or health-related subjects (c.f. Schiepe-Tiska et al., 2016a,b). Moreover, since we measured students’ goal orientations in second year in university, it might be possible that students already altered or adapted their goals to perceived expectations along with their academic studies. Future research should try to disentangle those potential effects with a longitudinal design and an alternative categorization of major subjects in university.

Yet, as a consequence from our study, intervention plans should perhaps focus on a better fit of social and communal goals with a STEM career to further increase the proportion of women entering and persisting in those fields. The current scientific discussion reflects on many approaches to helping women to persist and to succeed in STEM programs (c.f. van den Hurk et al., 2019; for a review). Recent efforts of universities and other stakeholders are focusing, for example, on mentoring programs for women in STEM fields and further on providing positive role models (e.g., Drury et al., 2011). These approaches might not only increase individual’s sense of belonging and feeling welcomed at an institution (e.g., Dasgupta, 2011; Ramsey et al., 2013) but also provide important information networks. Consequently, those approaches would pick up ideas to increase the support for students in STEM majors to provide help with academic difficulties and give constructive advice since these are often reported concerns, especially by students who switched from STEM to non-STEM majors (e.g., Seymour, 1992).

Furthermore, students economic situation (i.e., in this study: difficulties to pay for all expenses) was not relevant for the probability of profile membership, even though it was expected to be an indicator of further reasons to pursue a STEM career or university major, respectively, since the salaries in STEM occupations are on average relatively high (e.g., Oh and Lewis, 2011). Moreover, in this study, results showed that students in STEM majors were almost two times as likely in the profile of economic goals as compared to the profile of social goal orientation. However, our findings do not confirm the relevance of an interaction of STEM and economic situation for students’ goal orientations and further research is needed with different indicators of socio-economic background and more information on the financial situation of the students.

Our study is limited to a cross-sectional analysis: Even though the students were examined at two time points (first and second year in university), we do not have data with repeated measures of their goal orientation. It would greatly increase the interpretation of our findings if there were longitudinal measures of students’ occupational goal orientations. This would enable researchers to analyze not only the initial goal orientation—maybe even before entering tertiary education—but also to provide the analyses to examine if and how the goal orientations change over the educational years in university. It is plausible to assume that there are not only selection processes but also socialization processes that are relevant for university students’ professional goal orientations. Overall, the differences between the groups in our study were not as pronounced as we expected. Gender differences and differences of students in STEM or non-STEM majors were similar and more pronounced in the comparison of economic and social goal orientation profiles. Since previous research showed a more differentiated pattern of gender differences in subdisciplines within the area of STEM subjects (c.f. German Federal Bureau of Statistics, 2018; Gisler et al., 2018), future research might also focus on differentiating STEM fields in more detail.

In conclusion, our findings did not confirm differences in the professional goal orientations between women (and men) in STEM and non-STEM majors in tertiary education. However, women were more oriented toward social aspects of occupations, whereas men were more oriented toward economic aspects of occupations. Furthermore, students enrolled in STEM majors allocated more importance to economic goals than social goals. Intervention plans to increase the proportion of women in STEM fields in tertiary education and the labor market should, according to our findings, probably focus more on the congruity of students’ goal orientations with future career prospects of university degrees in STEM fields. Previous research showed that while boys are overall more interested in mathematics and information technology, girls are in fact interested in health-related occupations within STEM fields (Schiepe-Tiska, et al., 2016a,b). One potential approach might perhaps be to highlight the manifold occupations that a major in a STEM fields opens up.

## Ethics Statement

The analyses of this paper are a secondary analyses of data published previously (Blossfeld et al., 2011). Data sources used for the analyses were the cohort of first-year students (doi: 10.5157/NEPS:SC5:11.0.0) of the German National Educational Panel Study (Blossfeld et al., 2011). All students from this cohort gave informed consent to participate in the panel by providing their phone number for being contacted for telephone interviews after being informed about the purposes of the study. Specific information about the recruitment process can be found in the field report of the study (Steinwede and Aust, 2012). All data analyses were performed via a download access at LIfBi in Bamberg, Germany that provided a controlled privacy environment for data access. Furthermore, an ethics approval for the analyses was obtained by the local ethics committee.

## Author’s Note

This paper uses data from the National Educational Panel Study (NEPS): Starting Cohort First-Year Students, doi: 10.5157/NEPS:SC5:11.0.0. From 2008 to 2013, NEPS data was collected as part of the Framework Program for the Promotion of Empirical Educational Research funded by the German Federal Ministry of Education and Research (BMBF). As of 2014, NEPS is carried out by the Leibniz Institute for Educational Trajectories (LIfBi) at the University of Bamberg in cooperation with a nationwide network.

## Conflict of Interest Statement

The authors declare that the research was conducted in the absence of any commercial or financial relationships that could be construed as a potential conflict of interest.

## Author Contributions

IW performed the statistical analyses and drafted the manuscript. LE, TS and BD contributed to the conception of this study and discussed the results of the analyses and contributed to the manuscript and the revisions. All listed authors read and approved the submitted manuscript.
